# Modulating gene expression in breast cancer via DNA secondary structure and the CRISPR toolbox

**DOI:** 10.1093/narcan/zcab048

**Published:** 2021-12-22

**Authors:** Jessica A Kretzmann, Kelly L Irving, Nicole M Smith, Cameron W Evans

**Affiliations:** Laboratory for Biomolecular Nanotechnology, Department of Physics, Technical University of Munich, Am Coulombwall 4a, 85748 Garching, Germany; Munich Institute of Biomedical Engineering, Technical University of Munich, Boltzmannstraße 11, 85748 Garching, Germany; School of Molecular Sciences, The University of Western Australia, 35 Stirling Hwy, Crawley, WA 6009, Australia; School of Molecular Sciences, The University of Western Australia, 35 Stirling Hwy, Crawley, WA 6009, Australia; School of Molecular Sciences, The University of Western Australia, 35 Stirling Hwy, Crawley, WA 6009, Australia

## Abstract

Breast cancer is the most commonly diagnosed malignancy in women, and while the survival prognosis of patients with early-stage, non-metastatic disease is ∼75%, recurrence poses a significant risk and advanced and/or metastatic breast cancer is incurable. A distinctive feature of advanced breast cancer is an unstable genome and altered gene expression patterns that result in disease heterogeneity. Transcription factors represent a unique therapeutic opportunity in breast cancer, since they are known regulators of gene expression, including gene expression involved in differentiation and cell death, which are themselves often mutated or dysregulated in cancer. While transcription factors have traditionally been viewed as ‘undruggable’, progress has been made in the development of small-molecule therapeutics to target relevant protein–protein, protein–DNA and enzymatic active sites, with varying levels of success. However, non-traditional approaches such as epigenetic editing, transcriptional control via CRISPR/dCas9 systems, and gene regulation through non-canonical nucleic acid secondary structures represent new directions yet to be fully explored. Here, we discuss these new approaches and current limitations in light of new therapeutic opportunities for breast cancers.

## INTRODUCTION

**Figure 1.**
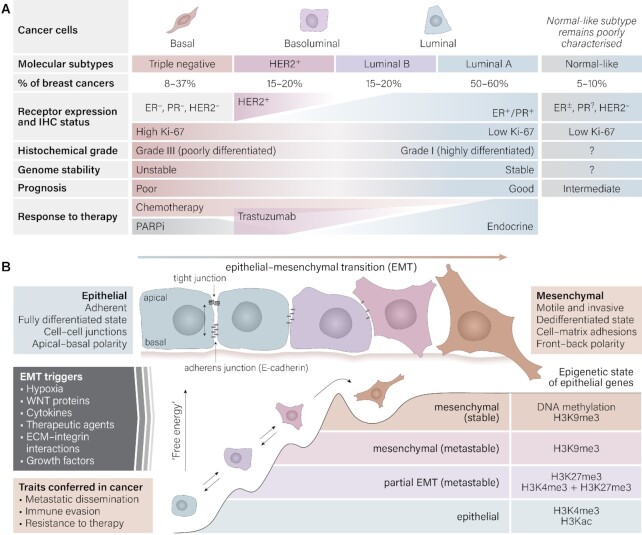
(**A**) Characteristics of breast cancer subtypes, based on collated data (241–245); ER, estrogen receptor, PR, progesterone receptor, PARPi, poly(ADP-ribose) polymerase (PARP) inhibitor. (**B**) Epithelial–mesenchymal transition showing key properties of epithelial and mesenchymal phenotypes, triggers and traits conferred by EMT in cancer. Adapted with permission from Springer Nature: Tam, W.L. and Weinberg, R.A. The epigenetics of epithelial–mesenchymal plasticity in cancer (2013). *Nat. Med*., **19**, 1438–1449 (246).

Breast cancer recently surpassed lung cancer as the most commonly diagnosed cancer, with over 16 million people currently living with breast cancer worldwide (1,2). Breast cancer is a disease with many faces, owing to genomic instability and aberrant transcription patterns, which contribute to a high level of heterogeneity. Existing therapies target various points in the breast cancer pathway, from the initial stage of preventing oncogenesis in high-risk individuals to addressing therapeutic resistance in advanced metastatic scenarios. As it stands, there are no ‘go-to’ therapeutic options which are reliable and effective for treating breast cancer in all its variants. Instead, current clinical practice typically divides breast cancer into five intrinsic subtypes: Luminal A, Luminal B, HER2-enriched, Basal-like and Normal-like, on which basis different treatment strategies may be chosen. Key characteristics of the different breast cancer subtypes are shown in Figure 1A. Across all subtypes, while the initial response to therapy is often positive, recurrence is common; in fact, the majority of breast cancer-related deaths (∼90%) are due to invasion and metastasis (3,4). For hormone receptor-positive subtypes in particular, there is a significantly elevated risk of recurrence in the following decades (5,6). Understanding how and why tumor cells leave the primary tumor site, acquire invasive properties, and colonize metastatic sites are therefore critical areas of research in breast cancer.

### Epithelial–mesenchymal plasticity

Epithelial–mesenchymal plasticity (EMP) is a key mechanism that facilitates breast cancer recurrence and metastasis. The epithelial-to-mesenchymal transition (EMT) describes the process by which tumor cells adopt a mesenchymal phenotype, becoming more invasive and motile (Figure 1B). Often, the driving force fueling EMP is the aberrant expression of an array of developmental transcription factors (TFs), collectively termed EMT-TFs (7). These EMT-TFs are responsible for the maintenance of a proliferative, undifferentiated phenotype during development and tissue regeneration, but their dysregulation in cancer can drive a gain of stemness, drug resistance, and increased invasive potential. Importantly, EMP is not regulated by an all-or-nothing switch, but rather a complex interplay of gene expression patterns, resulting in a continuum of intermediate cellular states (8–10). EMP is an epigenetically regulated and potentially reversible process (9,11,12), offering hope that with the correct therapeutic intervention, cells could transition back to a differentiated epithelial state or be re-sensitized to therapy.

Reducing the impact of EMT and re-sensitizing tumor cells to therapy relies on restoring a ‘normal’ profile of gene expression. Gene expression in breast cancer, just as in EMP, is highly plastic and affected by both mutations and epigenetic mechanisms. The regulation of gene expression is a circuitous process mediated by transcription factor activity and chromatin organization, which altogether modulate the ability of gene promoters to recruit RNA polymerases. Historically, TFs have been viewed as ‘undruggable’ due to the difficulty in designing small-molecule therapeutics to mediate specific protein–DNA or protein–protein interactions. More recent attempts have instead targeted the active sites of enzymes or have been designed for allosteric modulation of protein–protein interactions. Small-molecule drugs have also been developed to modulate the epigenetic state of the cancer cells by correcting DNA methylation, histone acetylation and histone methylation, for example. However, these agents all demonstrate low specificity and selectivity, and affect expression of a large proportion of genes, which ultimately leads to dose-limiting toxicity in patients (13). Furthermore, these approaches largely attempt to correct overexpression of oncogenic TFs, which is generally easier than attempting to upregulate dormant or silenced tumor-suppressor TFs, limiting the overall scope of potential targets.

The role of EMT in promoting cancer cell invasion, metastasis, and stem-like characteristics is well documented in several types of cancer, including breast cancer. Clinical samples of high-grade breast cancers have often been found to contain cells which express EMT program molecular signatures, particularly in tumors associated with poor patient prognosis (14,15). The evidence suggests that these tumors contain cells which have progressed through ‘partial EMT’ with the acquisition of some mesenchymal markers, while also retaining particular epithelial characteristics. These highly heterogeneous intermediate phenotypic states arise from numerous interactions between epigenetic modifications and transcriptional regulators (9,16).

### Epigenetic modifications are regulators of gene expression and EMP

Epigenetic modifications include histone acetylation, histone methylation and DNA methylation, which ultimately modulate chromatin accessibility, and together define a cell’s epigenetic profile. Relationships between epigenetic modifications and transcriptional activity are complex and depend highly on the additional context in which they are placed, and have been described comprehensively elsewhere (17–19). Epigenetic marks across the genome are highly altered in malignancies when compared to healthy tissue, and even differ between malignant cells across the EMP spectrum.

The progression of breast cancer through TF deregulation is complex, with many underlying shifts in epigenetic regulation and TFs acting in concert to direct the progression of cancerous cells through processes such as EMT. As demonstrated in Figure 1B, various histone modifications are gained during EMT, enabling a ‘bivalent’ epigenetic state of epithelial genes. However, the completion of EMT and stabilization of the mesenchymal phenotype requires epithelial genes to be repressed for extended periods of time. This is effected through highly stable DNA methylation (DNAme) near gene promoters, which causes gene silencing, and which is inherited with high fidelity over cell divisions (9). Stable epigenetic repression drives malignant cells toward a mesenchymal phenotype and toward therapeutic resistance (9,11,12).

EMT is driven by an orchestra of EMT-TFs, including SNAIL, SLUG, ZEB1/2 and TWIST. These EMT-TFs are involved in the regulation of E-cadherin, N-cadherin and vimentin, as well as a range of tumor suppressor genes. Loss of *CDH1* (encoding E-cadherin) expression is a hallmark of EMT and can occur through either promoter methylation or transcriptional repression of *CDH1*. E-cadherin is both a tumor suppressor gene and a critical component in cell adhesion junctions (20–22). EMT-TFs themselves drive widespread gene expression changes through cooperation with multiple epigenetic modifiers. For example, SNAIL (encoded by the *SNAI1* gene) cooperates with G9a, a H3K9 methyltransferase, to induce further recruitment of histone deacetylases (HDACs) and finally DNA methyltransferases (DNMTs), resulting in promoter DNAme and subsequent stable repression of E-cadherin (9,23). Indeed, stable silencing of E-cadherin by DNAme is found in claudin-low breast cancers, one of the most mesenchymal subtypes. In a different example, TWIST has been shown to recruit DNMT3B to the estrogen receptor 1 (*ESR1*) promoter, resulting in DNAme and loss of ER expression, and the progression of breast cancers that are ER-negative and hormone-resistant (24).

**Figure 2.**
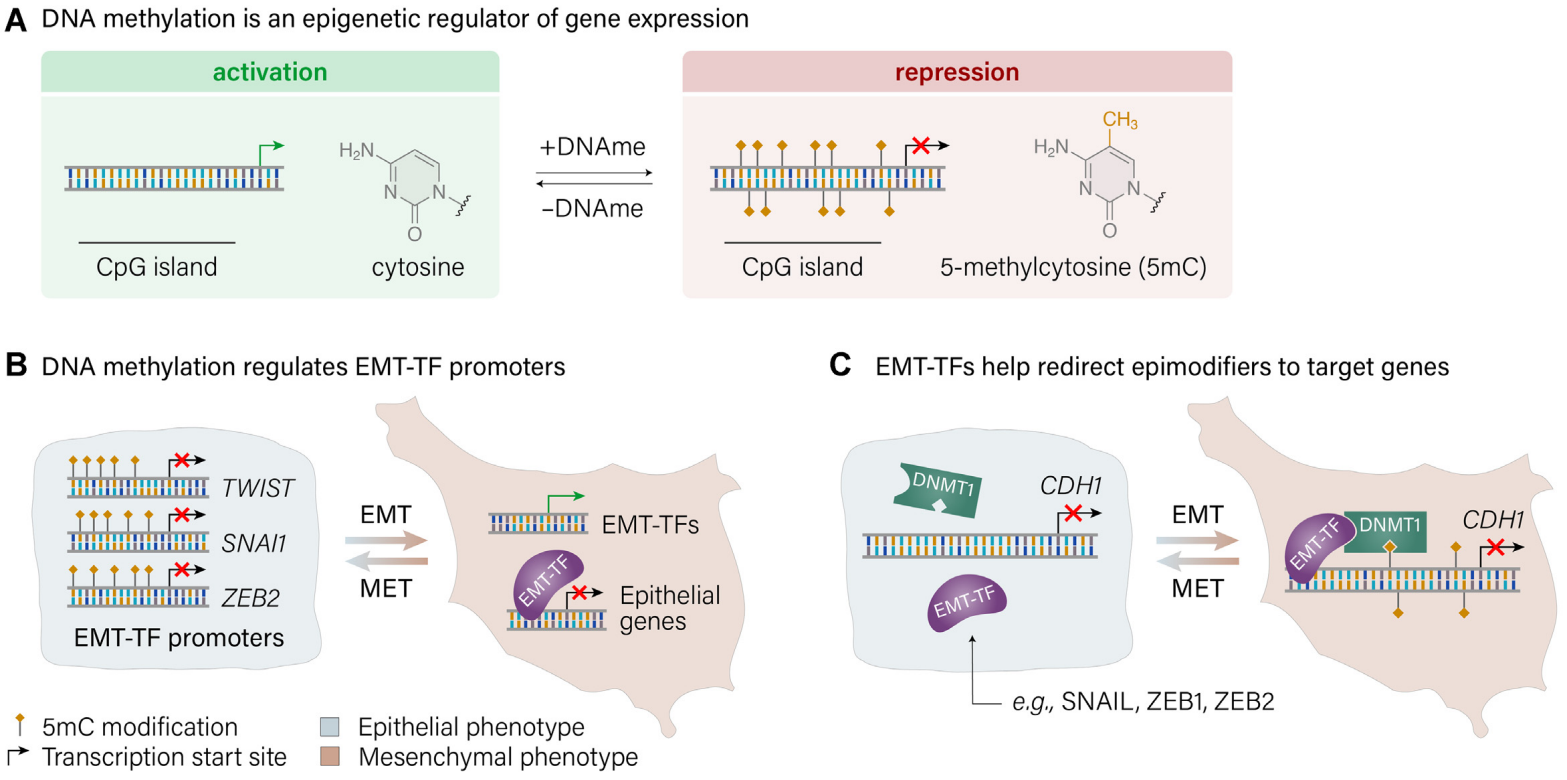
Key roles of DNAme in regulating gene expression in EMT. (**A**) DNA methylation of gene promoter CpG islands acts to repress gene expression. Methylation marks are deposited by DNA methyltransferase (DNMT), and removed by ten-eleven translocation (TET), families of enzymes. (**B**) Genes encoding EMT-TFs including TWIST, SNAIL (*SNAI1*) and ZEB2 are silenced by promoter methylation in epithelial cells. When these EMT-TFs are activated by EMT, they repress expression of epithelial genes, causing loss of cell adhesion and other epithelial phenotype characteristics. (**C**) EMT-TF expression can recruit epigenetic modifiers such as DNMT enzymes to methylate promoters of epithelial genes, resulting in stable and long-term repression. Adapted with permission from Elsevier: Skrypek, N., Goossens, S., De Smedt, E., Vandamme, N. and Berx, G. (2017) Epithelial-to-Mesenchymal Transition: Epigenetic Reprogramming Driving Cellular Plasticity. *Trends Genet. TIG*, **33**, 943–959 (31).

Hypermethylation of promoter CpG islands is a hallmark of cancer progression and typically correlates with transcriptional repression of the associated gene, as illustrated in Figure 2A (25). However, it is important to note that the exact role and relationship between methylation and gene expression remains unresolved, and seems to depend highly on the specific context (26). Promoter hypermethylation is commonly associated with a decrease in transcriptional activity and thought to alter the recruitment of regulatory proteins to the underlying DNA sequence, subsequently blocking transcriptional activation. Alternatively, methylation can provide binding sites for methyl-binding proteins which can act to mediate gene repression through their interactions with HDACs (27–29). In breast cancers, genomic stability and epigenetic silencing of tumor suppressor genes is often associated with both DNA hypermethylation in combination with aberrant histone modification.

The relationships between epigenetic modifications and EMT-TFs are less well understood. For example, promoter hypermethylation of *TWIST1* has been observed but has not been directly correlated with gene repression in breast cancer, although the relationship has been established for other cancers (30). There is evidence, however, that DNAme within regions of *SNAI1* (SNAIL) and *SNAI2* (SLUG) are correlated with gene repression, in addition to promoter hypermethylation of *ZEB2* (31). Therefore, there is evidence to suggest that some EMT-TFs are themselves regulated through DNAme, as shown in Figure 2B.

EMT can be driven by different factors depending on the cellular context. For example, EMT in itself does not always lead to an increase in stemness; however, it can do so when driven by stable expression of pluripotency factors, such as *SOX2*. SOX2 is a developmentally important TF which impacts the progression of breast cancers. *SOX2* is not expressed in most normal adult tissues but is expressed in ∼43% of basal-like breast cancers (32). *SOX2* expression is associated with an increased stem-like phenotype, increased invasiveness and metastatic potential, and therapy resistance (33,34). Interestingly, Leis *et al.* suggest that the increased stemness through reactivation of *SOX2* in early stage breast cancer may explain the high tumor heterogeneity (35). DNAme within the promoter and enhancer region functions as an epigenetic switch and forces cells into differentiation pathways. Stolzenburg *et al.* demonstrated that stable introduction of *de novo* DNAme in the *SOX2* promoter enabled long-lasting oncogenic repression in an MCF7 xenograft mouse model of breast cancer (7).

### DNA methylation stabilizes the epigenetic profile of breast cancer

Aberrant DNAme patterns are implicated in the initiation, promotion, and progression of breast cancers. Breast cancers demonstrate DNA hypermethylation at promoter regions of tumor suppressor genes and hypomethylation in regulatory regions of oncogenes. Aberrant DNAme patterns have also been associated with the acquisition of drug resistance, which is a major obstacle in breast cancer treatments and accounts for approximately 90% of unsuccessful treatments (36,37). Within human genomes, methylation predominantly occurs at cytosine (C) followed by a guanine (G) residue, termed CpG dinucleotides (38). CpG dinucleotides themselves are low in density throughout the genome, but cluster together in what are known as CpG islands within human gene promoter regions (38–40). Generally human promoter CpG islands are unmethylated, but can accumulate methylation patterns during development, or during other processes such as carcinogenesis (41,42).

Interestingly, DNA methylation can be directed through various stages of histone modifications. For example, histone methyltransferase EZH2 typically catalyzes tri-methylation of histone H3 but can additionally control methylation through direct contact with DNA methyltransferases (43–46). In a similar way, EMT-TFs can also recruit DNA methyltransferases (Figure 2C). Over 100 genes have been observed to display promoter hypermethylation in breast cancers, with many of them involved in critical cell processes such as cell cycle regulation, genome integrity, apoptosis, metastasis and invasive potential (25,37,47,48).

Gene silencing through promoter hypermethylation is an important mechanism in the development of both carcinogenesis and tumor resistance. For example, promoter CpG island hypermethylation is observed in *BRCA1*; the wild-type *BRCA1* tumor suppressor gene is commonly silenced in nonfamilial breast and ovarian cancers (25) and is associated with increased cancer invasiveness and mortality (49). Additionally, *BRCA1* promoter methylation is found in 11–31% of sporadic breast cancers and 20–60% of sporadic TNBCs (50,51). In a further example, ER is a critical transcription factor regulating events important to mammary gland development, such as cell division (52). In general, high ER expression correlates positively with better clinical outcomes and recovery, but resistance is frequently acquired throughout the therapeutic course (36). ER expression may be lost through hypermethylation of the ER gene promoter (24) but can be reinduced in an ER-negative cell line (MDA-MB-231, TNBC) by inhibiting DNMT activity (53).

### Summary

Gaining control over EMP and related epigenetic changes could lead to important new therapeutic approaches designed to limit resistance, metastasis and recurrence in breast cancer. Moving forward, researchers not only have an increased understanding of the complex regulatory roles and relationships between TFs but also new approaches that can be used for gene regulation. Both DNA secondary structure and an ever-expanding site-specific gene-modulation toolkit, through the Clustered Regularly Interspaced Short Palindromic Repeats and CRISPR-associated protein (CRISPR/Cas) genome engineering platform, can be used to modulate gene expression. Together, these approaches represent new ways to target the aberrant expression of TFs for breast cancer treatment.

## NON-CANONICAL DNA SECONDARY STRUCTURES AS MODULATORS OF GENE EXPRESSION

Global chromatin architecture is tightly regulated at the epigenome level, where specific epigenetic marks promote relaxed or condensed chromatin states, in turn coordinating the transcriptional accessibility of genomic regions. The dysregulation of global epigenetic marks at both the DNA and histone level significantly disrupts this regulatory mechanism, promoting disease-associated aberrant transcript levels. Nucleosome-depleted regions can permit the formation of non-canonical DNA secondary structures which provide an additional layer of control over local DNA accessibility, modulating TF binding and transcriptional activity (54,55).

Non-canonical DNA conformations have revealed themselves as regulators of gene expression and disease progression, whether through direct or indirect effects on transcription. In this section, we will discuss non-canonical DNA secondary structures in the context of gene expression and their formation in cancer genomes, with a focus on modulation of TF expression and transcriptional activity.

### G-quadruplexes and i-motifs are non-canonical DNA secondary structures

G-quadruplexes (G4s) and i-motifs are two highly dynamic non-canonical nucleic acid secondary structures which have emerged as important biological elements, with apparent regulatory roles over key processes such as replication and transcription (56–58). G4s and i-motifs can form at both the DNA and RNA levels, but the focus of this review will be at the DNA level. Since dysregulated TF–DNA interactions are key drivers in breast cancer progression, understanding the relationship between G4s, i-motifs, and TFs will help to uncover new therapeutic strategies for inhibiting EMP and modulating the epigenetic regulation of gene expression. Here, we discuss G4 and i-motif DNA secondary structures and their therapeutic potential within breast cancers.

**Figure 3.**
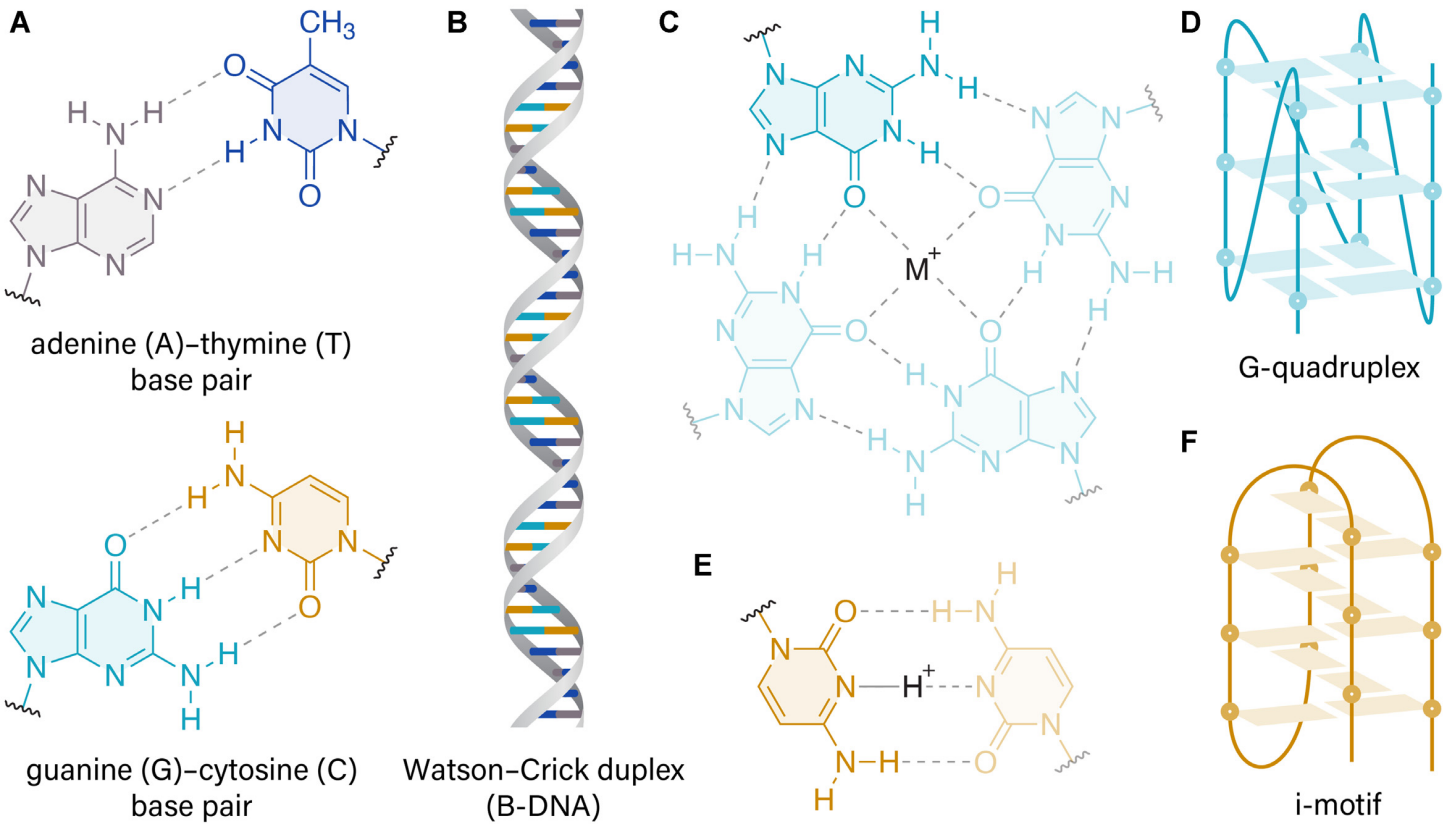
Schematic representation of DNA secondary structures. (**A**) Adenine–thymine and guanine–cytosine canonical Watson-Crick base pairing and (**B**) schematic representation of B-form DNA. (**C**) Planar G-tetrad structure formed through the self-association between four guanine bases via Hoogsteen hydrogen bonding, with a central monovalent cation shown within the center of the tetrad structure. (**D**) Schematic representation of an intramolecular G-quadruplex structure containing three stacked G-tetrads. (**E**) Hemi-protonated cytosine–cytosine base pairing, highlighting the N3 protonation of the cytosine ring. (**F**) Schematic representation of an intramolecular i-motif structure, through the self-association of several intercalated C···C^+^ base pairings.

G4 formation occurs within certain G-rich sequences containing several short tracts of contiguous guanine bases separated by intervening nucleotides (59–62). In contrast to the well-known Watson–Crick base pairing where a guanine base-pairs with cytosine (Figure 3A,B), four guanine nucleotides self-associate through Hoogsteen hydrogen bonding to form a G-tetrad (Figure 3C). Multiple G-tetrads π-stack upon each other to form a G4 with loops arising from intervening nucleotides between adjacent G-tracts (Figure 3D) (63,64). G4s can be intramolecular where they form from a single DNA strand, or intermolecular where they consist of two or four separate DNA strands (56,57). In contrast, i-motif structures form within certain C-rich sequences through hemi-protonated cytosine–cytosine, or C···C^+^, base pairings (Figure 3E). These hemi-protonated cytosine–cytosine base pairs intercalate upon each other resulting in the i-motif structure (Figure 3F) (65–67). Like G4s, i-motifs can be intra- or inter-molecular, arising from single or two/four separate DNA strands, respectively. In most cases, G4s and i-motifs can be resolved by helicases during processes such as DNA replication and transcription, and their stability varies depending on the extent of base stacking and intervening loop lengths. While G4 stability can be affected by type and concentrations of cations, i-motifs are more sensitive to pH alterations (68,69). In general, i-motifs have been much less studied within the cellular context than G4s (70,71).

Many oncogene promoters contain sequences capable of forming G4s or i-motifs. A greater abundance of G4s is observed in various cancer cell types compared to their non-tumorigenic counterparts (72,73), with G4 formation occurring at a higher density in promoters of actively transcribed genes, nucleosome depleted regions and being colocalized with RNA polymerase II and trimethylated histone (H3K4me3), which is a histone modification associated with active genes (74). Of significant interest to breast cancer are regulatory G4 and i-motif elements within the promoter regions of key oncogenes including *BCL-2* (56,75–79)*, c-MYC* (77,80–84), *KRAS* (85–88), *hTERT* (89–92), *PDGFR-β* (93,94), *c-KIT* (95–102), *RET* (103,104) and *VEGF* (105). Interest in the biological relevance of DNA secondary structures initially arose due to computational studies, which demonstrated sequences with the propensity to form G4s or i-motifs are overrepresented within regulatory regions of the human genome (59–62,69,106). Numerous studies have attempted to determine the mechanisms by which promoter G4 or i-motif structures shape gene expression, but no hard-and-fast rules have yet emerged. Multiple factors need to be considered such as positioning of the G4 on the template versus non-template strand and overlap with TF binding sites (107–111).

Promoter G4 structures were initially viewed as suppressive elements, physically blocking the accessibility and progression of transcriptional machinery (112,113). However, the first G4-ChIP-seq in human cells in 2013 found a strong association of endogenous G4s with the promoters of highly transcribed genes, linking G4s with increased transcriptional activity (74,114). Furthermore, a recent study demonstrated that endogenous G4s coincide with prominent TF binding sites in human chromatin, particularly in the promoters of highly transcribed genes (111). This finding was consistent across two different cell lines with distinct G4 landscapes, suggesting that G4 binding is a general property of certain TFs (115). However, a recent study using the G4-CUT&Tag method demonstrated that ligand-induced G4-stabilization actually reduced transcriptional activity by inhibiting binding of TFs (116). Thus, while endogenous G4s have been previously associated with enhanced transcription, G4-stabilizing ligands may actually downregulate or suppress transcription (116). Further investigation to see if this pattern is consistent over different ligand concentration, ligands, G4s and genes will be an interesting future direction.

Under Watson–Crick base pairing rules, G-rich regions of the genome are complemented by C-rich regions on the opposing DNA strand, and vice versa. Sites of G4 and i-motif formation should therefore exist at the same genomic loci on opposite strands, with an apparent potential for these two structural motifs to form simultaneously. However, increasing evidence indicates this does not occur (81,117–118). G4 and i-motif formation are altered during cell cycle progression, with G4 formation most prevalent in S phase, and i-motifs peaking at late G1 phase (70,114). Using molecular tweezers to measure torsional stress and extension forces, Sutherland *et al.* demonstrated that the *c-MYC* promoter sequence forms mutually exclusive G4 and i-motif structures in double-stranded DNA (81). It has been proposed that these complementary structures may act as a ‘molecular switch’ for gene expression (56,81,86). The observation that global stabilization of G4 structures in human cells reduces the abundance of i-motifs, and vice versa (71), indicates that the molecular switch mechanism may be a genome-wide phenomenon. Together, these findings point towards a biological ‘on/off’ switch which could potentially be exploited with future therapeutics.

### DNA secondary structures contribute to genomic instability

Increasing evidence suggests that DNA secondary structures may contribute to aberrant oncogenic TF levels as well as influencing epigenetic disease states (54–55,119–121). The aberrant formation of G4 structures can result in epigenetic instability due to hindered biological processing of DNA. For example, unresolved G4 structures can physically block transcription, result in the accumulation of DNA replication errors, or cause double-strand breaks (122–125). Sites of highly stable DNA secondary structure formation may therefore act as mutational hotspots. Mutations localized at G4 formation sites within promoter regions could further influence cancer susceptibility by altering transcriptional activity, protein interactions, DNA secondary structure formation and epigenetic patterns (89,123,126–130). Mapping the genome-wide occurrence of G4 structures using quantitative G4-ChIP-seq in 22 patient-derived tumor xenograft breast cancer models revealed differential G4 formation sites were significantly enriched in single-nucleotide variants (SNVs). SNV enrichment highlights a potential role for G4s in the development of breast cancer point mutations (131). Differential G4 formation sites were also enriched in the promoters of highly amplified genes and correlated with differential TF binding sites and differential TF expression levels. Interestingly, the 45 most common breast cancer driver regions characteristic for copy-number aberration-induced gene expression alterations were also linked to such G4-forming and TF-binding enriched sites. Further research into the link between DNA secondary structure formation and mutation may provide insights into how early cancer cells acquire disease-associated states.

### G-quadruplex and i-motif promoter structures with therapeutic potential in breast cancer

It is interesting to note that each of the six hallmarks of cancer has been previously associated with oncogene promoter G4 or i-motif structures: self-sufficiency (*c-MYC* and *KRAS*), limitless replication (*hTERT*) (89–92,132), evasion of apoptosis (*BCL-2*) (56,77–79,133,134), sustained angiogenesis (*VEGF*) (105,135–139), invasive and metastatic potential (*PDGFA*) (140) and insensitivity to anti-growth signals (*pRb*) (66,141). For a more comprehensive review of this topic we direct the reader to Brooks *et al.* (66). The links between some important examples of promoter G4-/i-motif-mediated transcriptional control in the context of potential therapeutics for breast cancers are presented below.

#### Links between DNA secondary structure and cell proliferation genes

Breast cancer cells can acquire the ability to continuously proliferate through various molecular pathways. Genes including *hTERT*, *HER2* (*ERBB2*), *HRAS*, *KRAS* and *c-MYC* confer increased proliferative capacity in breast cancer (142,143). Each of these genes has been linked to silencing in cancer through DNA secondary structure formation, as discussed below. In particular, it is well established that the protein products of the *RAS* family (144) and *c-MYC* (145) are essentially undruggable due to a lack of targetable domains within the protein.

Telomerase, the enzyme essential for maintaining telomere length, is usually silenced in somatic cells but is expressed in ∼90% of cancers, including breast cancers (146). The *hTERT* core promoter region contains two end-to-end stacked G4 structures connected by a hairpin loop that acts as a silencing element (89,90). The G4-forming region of *hTERT* is mutated in many cancers resulting in loss of G4 formation and subsequent *hTERT* activation. Kang *et al.* and Song *et al.* demonstrated that small molecule ligands which refold the hTERT promoter G4, even in the presence of these mutations, result in transcriptional repression of hTERT and cancer cell death (89,90).

*HER2* is overexpressed in ∼25% of human primary breast cancers across all subtypes. The HER2 promoter contains a G4-forming sequence which is the binding site for several TFs (147). When folded, the *HER2* promoter G4 element acts to block transcription resulting in repression of *HER2* (148). Downregulation of *HER2* levels is a potential therapeutic target and has been demonstrated within breast cancer cells via a luciferase reporter assay whereby stabilization of the *HER2* promoter G4 structure downregulated *HER2* expression at both the mRNA and protein levels (148).

Both the *KRAS* and *HRAS* promoter regions contain stable G4 and i-motif structures. G4 formation represses expression of each of these genes (85,86,149–152). In *KRAS* and *HRAS*, promoter G4s colocalize with binding sites for TFs such as MYC Associated Zinc Finger Protein (MAZ) and Specificity Protein 1 (SP1) (109,153). Binding of MAZ activates expression of *KRAS* and *HRAS* (152,154). Interestingly, the *HRAS* oncogene promoter contains two neighboring suppressive G4s, and both of which can be resolved by MAZ, restoring *HRAS* expression (150,151). Similarly, i-motif formation in *HRAS* and *KRAS* recruits heterogeneous nuclear ribonucleoprotein (hnRNP) family members hnRNP A1 and hnRNP K respectively, which unfold the i-motif structure resulting in increased oncogene expression (86,151). Breast cancers tend to lack the *RAS* promoter mutations which are commonly observed in other cancer types (155). However, the above TF-regulated G4/i-motif molecular switch mechanism influencing *KRAS* and *HRAS* expression can rationalize *RAS* activity in the absence of mutation. Accordingly, destabilization of hnRNP protein/i-motif interactions or stabilization of promoter G4 structures has been reported to significantly inhibit *HRAS* and *KRAS* expression (85,154,156).

*c-MYC* is the primary oncogenic driver of cancer gene expression programs in a broad spectrum of cancer types, and is an inducer of EMT (145). The G4- and i-motif forming nuclease hypersensitive element III (1) (NHEIII_1_) of the *c-MYC* promoter region is the principal regulator of *c-MYC* expression (80). The interplay between single-stranded DNA binding proteins and structure-specific resolving and/or stabilizing proteins determine structure formation capabilities pivotal for downstream *c-MYC* expression levels (81). Sutherland *et al.* demonstrated how the TF SP1 influences *c-MYC* expression through interactions between i-motif structure, hnRNP K and nucleolin (81). Ligand-based stabilization of the *c-MYC* G4 or i-motif has been shown to downregulate *c-MYC* expression (80,157).

#### Interplay between DNA secondary structures and EMT-TFs in breast cancer

Two crucial EMT-TFs, namely ZEB1 (*ZEB1*) and SNAIL (*SNAI1*), have the ability to form G4 structures within their respective gene promoter regions (158,159). Through recent independent studies, both the *ZEB1* and *SNAI1* promoter G4 elements have been shown to suppress transcription, whereas high expression levels of each of these genes were associated with a lack of G4 formation. Together, these studies provide some initial insight into relationships between EMT-TFs and promoter DNA secondary structures. Wang *et al.* demonstrated that G4 formation in the *SNAI1* promoter acts to repress expression and found a high number of mutations in this G4 region which affect G-tract lengths or distributions, potentially relating changes in *SNAI1* expression to changes in G4 formation (158). While the *SNAI1* promoter G4 has yet to be targeted with small molecules, stabilization of the *ZEB1* G4 structure was effective in downregulating *ZEB1* expression levels and inhibiting cell migration (159).

TFs such as hypoxia inducible factor 1 alpha (HIF1α) and MYB (encoded by the proto-oncogene *c-MYB*) are both strongly associated with the upregulation of EMT-TFs (160–162). The levels of both HIF1α and c-MYB TFs are mediated via suppressive G4 formation within their corresponding gene promoters (163,164). Stabilization of a *HIF1a* promoter G4 inhibits gene transcription by disrupting binding of the transcription factor AP2 to the promoter G4 (163), while G4 formation in the *c-MYB* promoter alters TF biding, where a complex between the G4 and the MAZ TF downregulates *c-MYB* expression (164). G4-mediated expression of both the c-MYB and HIF1α TFs therefore represent attractive targets for future research.

#### DNA secondary structure can modulate TF activity

Spiegel *et al.* and Lago *et al.* recently demonstrated an enrichment of TF binding at G4 forming sites within promoters across the human genome (111,115), which correlated with increased transcriptional activity when compared to promoter regions void of G4 structures. In fact, the interaction between DNA secondary structures and TFs is bidirectional. Just as G4 and i-motif formation affects TF binding, TFs themselves can also influence G4 and i-motif structures by unwinding, or by binding to DNA and preventing structures from forming (86,115,122,151,165). TF-governed formation of DNA secondary structures is, therefore, highly susceptible to aberrant TF levels associated with breast cancer progression. It is evident that a complex relationship exists between TF-mediated G4 stability and G4-dependent TF binding.

TF recognition of G4s or i-motifs may provide a unique opportunity for modulation of gene expression. For example, manipulation of TF binding has been demonstrated in relation to the i-motif-dependent hnRNP K regulation of *KRAS* expression, where the inhibition of i-motif formation prevented hnRNP K binding (86). Similarly, the stabilization of the *HIF1α* promoter G4 structure results in the displacement of AP-2 (activator protein 2) TF binding, leading to the subsequent reduction in *HIF1α* expression (163). The ability to modulate TF binding in such a precise manner is of interest when targeting promiscuous TFs, which often display both cancer promoting and cancer suppressing activities at different genomic loci. One example is SP1, which can act to dysregulate multiple target genes within breast cancer cells (83,115). Therefore, modulation of SP1 activity would therefore require locus-specific precision.

DNA–protein interactions play a crucial role in the coordination of chromatin states and distal interactions, an associated yet additional layer of genomic regulation on top of transcriptional activity (166). The observation that G4 and i-motif structures are found within nucleosome-depleted genomic regions suggests that these structures may influence chromatin dynamics (74). Therefore, understanding the mechanisms influencing the binding abilities of the previously mentioned TFs, in addition to chromatin regulating proteins, is an area of increasing interest. *ZEB1* repression via promoter G4 stabilization provides one recent example of the relationship between chromatin remodeling proteins and promoter G4 structures. Stabilization of the *ZEB1* promoter G4 displaces nucleolin, which impedes SP1 co-factor binding, preventing the further recruitment of the histone acetyltransferase P300. This cascade alters the promoter’s architecture by shifting the chromatin into a closed (repressive) state, modulating transcriptional activity (159). A similar relationship has been observed for lysine-specific demethylase 1A (LSD1), which displays G4-mediated recruitment to the G4-bound TFs, telomeric repeat binding factor 2 (TRF2) and nucleoside diphosphate kinase 2 (NME2). G4-mediated LSD1 recruitment within the promoter regions of *p21* and *hTERT* promotes repressive histone marks resulting in gene silencing (167,168).

DNA sequences with the propensity to fold into secondary structures are implicated in DNAme density and deposition patterns, providing another mechanism by which G4 and i-motif structures impact the organization of chromatin (169,170). DNMT3A, DNMT3B and DNMT1 have high binding affinities for the 3D structure of folded G4s (171–173). Upon binding to a G4, DNMT1 loses enzymatic activity and physically blocks the surrounding DNA sequence from DNAme, thereby acting as local epigenetic regulatory element (171). Generally, G4 formation is negatively correlated with DNAme (173,174). Interestingly, however, breast cancer-associated hypermethylated sites display a threefold increase in G4-forming sequences. This observation suggests that while DNAme at sites of G4 formation is not biologically conserved, disease progression may act to alter this relationship (175). Comparison of DNAme patterns of 44 different i-motif forming sequences within a human breast cancer cell line (MCF7) and a non-tumorigenic mammary epithelial cell line (MCF10A) indicated 27% of i-motif sequences contained at least one 5mC, and of these, 83% were differentially methylated in MCF7 *vs* MCF10A, highlighting the differences in methylation between cancer and non-cancerous cell types (176).

At the molecular level, G4s tend to exhibit higher thermal stability when a DNAme modification is present within the sequence (177,178). Similarly, i-motif structures also tend to exhibit higher stability when a DNAme modification is present (176,179,180). However, the extent of methylation within an i-motif forming sequence has also been shown to be important, with hypermethylation destabilizing certain i-motif structures, such as the c-MYC i-motif (179). Furthermore, the position of the DNAme modification is also critical for the stability of certain i-motifs, such as the human telomeric hTelo i-motif (176). Unsurprisingly the change in structure stability is associated with altered binding capabilities of proteins. For example, aberrant DNAme modifications in the G4 forming sequence within the first exon of the hTERT oncogene enhances G4 stability, which in turn, impedes binding of the highly conserved CCCTC-binding factor (CTCF) TF, resulting in the oncogenic upregulation of hTERT expression (181).

In addition to chromatin state, G4-protein binding has been implicated in promoter-enhancer interactions, allowing chromatin looping either through G4-TF binding, or via long-range split G4 sequences that when facilitated by DNA looping can come together to form G4 structures. G4-dependent looping has been shown to be facilitated by the direct binding of the TF Yin Yang 1 (YY1) to promoter G4s, allowing DNA looping to occur between two bound YY1 sites (182). Disruption of G4 formation through helicase overexpression or CRISPR-gene editing diminished YY1-mediated DNA looping, highlighting the critical role of G4s in DNA looping. Disruption of YY1–G4 interactions via G4-stabilizing ligands resulted in altered gene expression not only of promoter G4-harboring genes but also of promoter G4-lacking genes through the dissociation of YY1 DNA looping with distal G4 structures. Interestingly, the YY1 promoter sequence itself is thought to be under the control of G4 formation, which is resolved within cancer cells due to elevated levels of the G4-resolving helicase G4R1 (165).

### Summary

While the exact, mechanistic roles of G4s and i-motifs in breast cancers remain to be determined, it is clear that both secondary structures contribute to the progression of cancer and EMT. Regulatory regions of oncogenes are enriched in G4 and i-motif structures and are implicated in aberrant gene expression patterns and genomic instability through physical blocking of transcriptional machinery, and through their interactions with various TFs, epigenetic modifiers and modifications. Overall, the evidence suggests that G4 and i-motif structures control gene expression patterns via a molecular switch mechanism, creating a potential new avenue for targeting therapeutics directly to DNA.

## TAKING CONTROL OF GENE EXPRESSION FOR BREAST CANCER THERAPY

Previous attempts at targeting aberrant TF expression have involved targeting protein–protein and/or protein–DNA interactions, which has been a difficult task for small molecules where the protein of interest is undruggable (i.e. lacks binding sites for small molecule interactions). Targeting TF expression at the genetic level, however, has recently become possible due to developments in the field of non-canonical DNA secondary structure targeting and CRISPR technology. These two approaches will be discussed below, along with a discussion of their current limitations.

### Modulating DNA secondary structure formation with small molecules

The unique structures of G4 and i-motifs enable the design of small-molecule therapeutics with preferential selectivity for G4s or i-motifs over the more ubiquitous B-DNA. Targeting G4s or i-motifs in gene regulatory regions compared to enzymes or proteins allows us to target genes regardless of the ‘druggability’ and/or copy number of the gene product present. Small molecule therapeutics targeting either G4s or i-motifs in breast cancer models are summarized in Table 1; however, it is important to note that there are other promising drugs available that modulate DNA structure that have not yet been tested in the context of breast cancer, such as RG260 (90), and are reviewed elsewhere in respect to different cancers (183). These small-molecule drugs are designed to either stabilize or destabilize the target structures, which modulates gene expression. To date, there have been many more small molecules developed for interaction with G4s than for i-motifs, owing to the more recent discovery of i-motifs as physiologically relevant structures (57,65,112,184–186), so we have focused the discussion towards small-molecules targeting G4s.

**Table 1.** Examples of G4 and i-motif targeting using small molecules with therapeutic potential in breast cancer

Compound	Secondary structure	Impact on gene expression and/or cellular fate	Reference
360A	G4 stabilizing	Reactivation of *p21* expression, increased sensitivity to doxorubicin in breast cancer cells	(167)
Pyridostatin	G4 stabilizing	Activation of innate immune genes in breast cancer cells	(247)
PhenDC3	G4 stabilizing	Activation of innate immune genes in breast cancer cells	(247)
RHPS4 and derivatives	G4 stabilizing	Inhibits telomerase activity, induces damage foci at telomeres and apoptosis in breast cancer xenografts	(248–250)
GTC365	G4 stabilizing	Represses *hTERT* expression, decreased telomerase activity and telomere length, promoted cell death/senescence in breast cancer cells	(89)
BRACO-19	G4 stabilizing	Reduced telomerase activity and telomere length leading to cellular senescence in breast cancer cells	(89,251–254)
TGP18	G4 stabilizing	*BCL-2* downregulation, marginal inhibition of TNBC tumor growth in mouse xenograft breast cancer model	(255)
CX-5461	G4 stabilizing	Inhibits replication forks and induces DNA damage, resulting in cell death in BRCA-deficient TNBC patient derived xenograft models, currently in clinical trials	(200,201)
Cβ	G4 stabilizing	Represses *HER2* transcription and expression in breast cancer cells	(148)
QN-1	G4 stabilizing	*c-Myc* downregulation, cell cycle arrest and apoptosis, tumor suppression in TNBC mouse model	(256)
BTC f	G4 stabilizing	*c-Myc* downregulation demonstrated in hepatocellular carcinoma cells, inhibited growth in breast cancer cells	(257)
Pyridyl bis-prolinamide ligand 1	G4 stabilizing	Simultaneous inhibition of *c-Myc* and *BCL-2* expression, leading to cell cycle arrests, apoptosis and DNA damage in breast cancer cells	(258)
BMVC4	G4 stabilizing	*c-Myc* downregulation and telomerase inhibition in non-small cell lung carcinoma cells, inhibited growth in breast cancer cells	(259)
tanshinone IIA derivative 4	G4 stabilizing	Repression of c-*Myc*, *KRAS*, *VEGF-a/b*, *BCL-2* and *Tel-26* expression, suppressed metastasis and angiogenesis of TNBC cells	(260)
PBP2	G4 stabilizing	*BCL-2* downregulation, increased apoptosis in breast cancer cells	(261)
NiP	G4 stabilizing	Telomere uncapping, DNA damage and degradation of telomeric 3′ overhang, promoted apoptosis of breast cancer cell stem cells, reduced expression of stem cell markers	(262)
TMPyP4	G4 stabilizing	Decrease in *hTERT* expression, inhibition of telomerase activity, suppressed growth rate of breast cancer xenograft model	(263,264)
3,8,10-trisubstituted isoalloxazines	G4 stabilizing	Downregulation of *c-kit* gene expression in breast cancer cells	(95)
IMC-48	i-motif stabilizing	Upregulation of *BCL-2* in breast cancer cells	(56)
IMC-76	i-motif destabilizing	Downregulation of *BCL-2* in breast cancer cells	(56,76)
PBP1	i-motif stabilizing	*BCL-2* upregulation, decreased apoptosis in breast cancer cells	(261)

**Figure 4.**
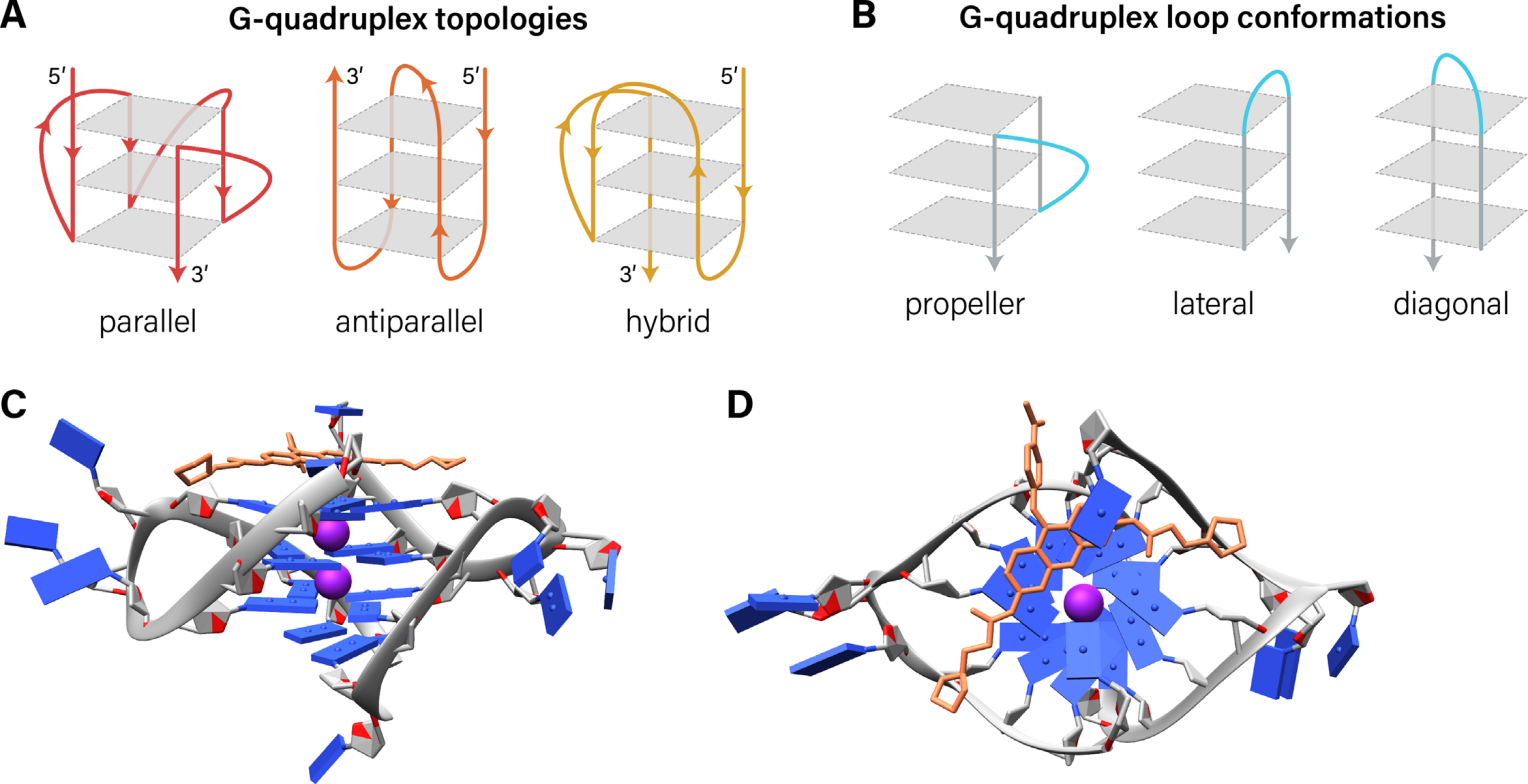
G-quadruplex topologies, loop conformations and example ligand binding modes. (**A**) G-quadruplex topologies for an intramolecular. (**B**) Loop conformations, highlighted in blue. (**C**) PhenDC3 ligand, shown in orange, interacts with a quadruplex via end-stacking and the grooves of this parallel G-quadruplex, PDB code: 3CE5. (**D**) Top-down view of the same structure as in (C).

All G4s have a common core of stacked G-quartets with a central ion channel (often stabilized by monovalent cations, such as Na^+^ or K^+^) and four grooves with varying dimensions which are determined by overall topology and loop geometries. G4s can differ in their topologies and loop geometries. Topology is based on the directionality of strands that make up the G-quartet core, classified as parallel, antiparallel or hybrid (187,188) (Figure 4A). The size and sequence of the loops determine the loop geometries. Loop geometries are categorized as propeller or double-chain-reversal loops, edgewise or lateral loops, diagonal loops, or V-shaped loops, as illustrated in Figure 4B (188,189).

Ligands that only target the G-quartet core show poor discrimination between different G4 structures, but selectivity can be improved by additional targeting of loops and grooves (190). However, design of a G4 ligand that demonstrates selectivity toward a single G4 still remains a major challenge. Overall, the majority of G4-binding small molecules share a few basic characteristics, possessing a heteropolyaromatic chromophore that is usually planar to enable π–π stacking interactions with a terminal G-quartet and one or more flexible side-chains with substituents featuring a cationic charge for interacting with the quadruplex grooves and loops (65,112,184,185). The majority of G4-targeting ligands stabilize the G4 structure and only a few ligands act to destabilize G4s (95,191–195). An example of a G4 structure interacting with PhenDC3 is depicted in Figure 4C,D; small-molecule therapeutics may stack upon the end of a G4 structure and/or interact with the distinctive loop structures of G4s (196).

There are several ways in which therapeutic targeting of DNA secondary structures can impact genomic stability and transcriptional activity. One of particular interest arises from the observation that genome-wide G4 or i-motif stabilization can result in the accumulation of DNA damage, leading to transient heterochromatin formation and transcriptional repression while damage is repaired (197,198). Breast cancer cells that have a compromised ability to repair DNA damage or properly unwind DNA during replication can be particularly sensitive to the stabilization of DNA secondary structures compared to their non-malignant counterparts. For example, cells with impaired homologous recombination repair pathways (which includes cells deficient in BRCA1 and BRCA2) display higher levels of genomic instability, cell cycle arrest and apoptosis following stabilization of G4 structures (199). In this scenario, stabilization of G4s blocks replication folds and induces single-stranded DNA gaps or breaks, where failure to repair this damage through the BRCA and non-homologous end joining pathways leads to cell death. Compound CX-5461 achieves specific toxicity against BRCA-deficient cancer cells through this mechanism. Importantly, CX-5461 resulted in dramatic tumor regression in polyclonal patient-derived xenograft models, including a TNBC tumor already pretreated heavily with other therapeutics (including platinum drugs), and a TNBC tumor derived from a patient who had been pretreated with anthracycline/taxane, and whose metastatic disease had minimal response to cisplatin. CX-5461 ultimately enables a new therapeutic option for aggressive cancers with BRCA deficiencies, including those resistant to PARP inhibition, and is in clinical trials (200,201).

Similarly, cells deficient in G4-unwinding helicases such as Fanconi anemia group J protein (FANCJ), which is commonly mutated in breast cancers, also prevents cells from resolving G4 structures, making G4-helicase deficient cells sensitive to secondary structure stabilizing treatments (122,202). Alternatively, G4-stabilizing molecules may increase susceptibility and enhance treatment efficiency when combined with traditional breast cancer chemotherapeutics. As an example, doxorubicin-resistant breast cancer cells can regain doxorubicin sensitivity after the addition of G4-stabilizing molecule 360A (167). Interestingly, Guilbaud *et al.* demonstrated that chromatin modifications which occur due to the stabilization of G4s can be inherited and persist even after the removal of the G4 stabilizing molecule (121), which suggests that transient DNA structure stabilization may be sufficient for a sustained therapeutic effect.

One of the major issues with traditional small molecule therapeutics for cancer treatments has been the severe off-target and side effects. Naturally, this also raises concerns about small-molecule therapeutics for targeting DNA secondary structures. In general, the selectivity in targeting DNA secondary structures in cancer cells stems from the (i) the high metabolism and protein synthesis requirement in cancer cells (203), and (ii) the observation that the majority of small molecules do not induce G4s, but stabilize the ones which have formed (which occur at a higher level in cancerous cells, making these cells more susceptible). Together, these two general methods, along with the ability of healthy cells to unwind structures and repair DNA damage, seems to convey a level of specificity. Whether this is enough is yet to be answered. To reduce off-target effects, molecules targeted for G4 and i-motif interactions must have high selectivity over binding to duplex DNA and RNA, which would lead to nonspecific toxicity. This requirement seems to be readily achievable through molecular design, where selectivity for the larger surface area of terminal G-quartet increases with ligand size and overall number of side chain substituents. Further, the design of macrocyclic and crescent-shape molecules inherently show low duplex-binding ability due to the physical shape which prevents them from binding in duplex DNA intercalation sites (187). Selectivity for a specific secondary structure in relation to the control of a specific gene is more difficult, and while there has been some promise in demonstrating preference over one specific structure or conformation (such as preference of the Na^+^-stabilized G4 over the K^+^-stabilized G4 in the human telomeric sequence (204)), so far there are no predictive rules to govern molecular design. However, targeting a specific G4 may not be necessary, instead, it may be beneficial to target multiple G4-mediated pathways concurrently. The G4-stabilizing ligand triarylpyridine 20A affects several biological pathways with multiple G4s involved, which suggests multiple G4-dependent inhibitory effects (205). An alternative method of targeting at the gene level, which does enable controlled targeting of specific genes, is CRISPR/Cas technology.

### Targeted regulation of transcription factors using CRISPR/Cas systems

**Figure 5.**
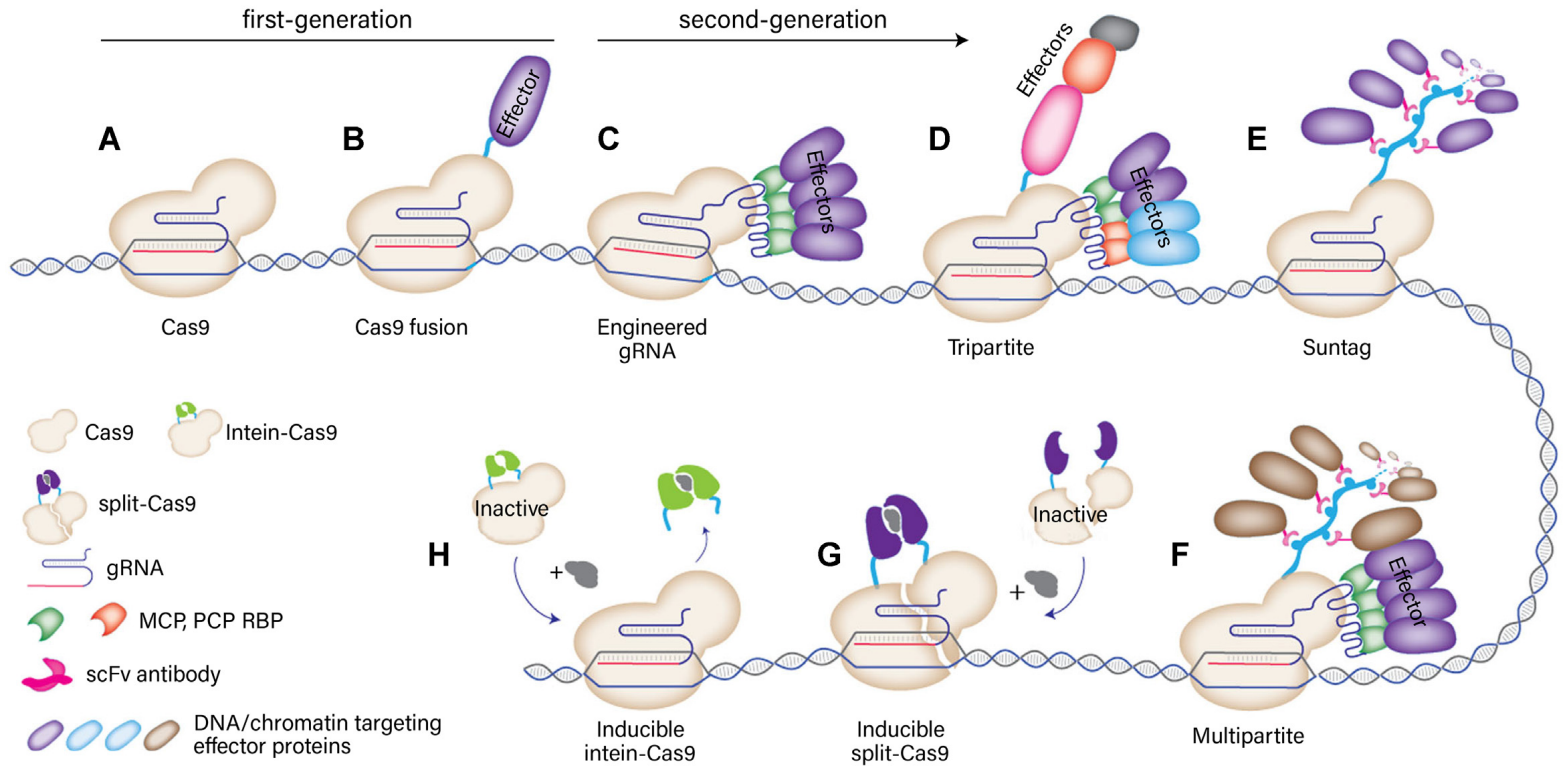
Schematic of the CRISPR/Cas9 toolbox. First-generation Cas9 systems include the (**A**) unmodified Cas9 system, and (**B**) the Cas9 enzyme fused to single effector proteins. Second-generation Cas9 systems (**C–H**) have evolved to enable either higher levels of modification, or more sophisticated mechanisms of controlling modification. The principle behind constructs aimed for higher levels of modification is to recruit multiple copies of effector proteins, which can be achieved through (C) engineering gRNA scaffold to include RNA aptamers (engineered sgRNA), (E) binding effectors along a repeating peptide array (Suntag), or (**D** and **F**) combinations thereof (tripartite and multipartite). Chemically inducible systems enable temporal control over the activity of the Cas9 fused effector proteins. (**G**) In the split-Cas9, each half of the Cas9 protein has to be induced to form the full, functional complex, while the intein-Cas9 approach (**H**) requires a protein segment to be excised after chemical induction. Adapted with permission from Adli *et al.* (2018) (207) under Creative Commons Attribution 4.0 International License (https://creativecommons.org/licenses/by/4.0/).

The ability to artificially modulate gene transcription could enable the reestablishment of normal gene regulation in diseased states, and program cell fate, behavior and tissue function. The ability to reprogram cells is fast becoming a relatively easy and accessible reality though CRISPR-based technology, and its quickly-expanding toolbox. CRISPR, standing for clustered regularly interspaced short palindromic repeat, together with CRISPR-associated proteins (Cas), are effectively an RNA-guided ‘homing’ device which can be used to target nearly any sequence of DNA with high specificity. The most widely used CRISPR/Cas system is CRISPR/Cas9, specifically the *Streptococcus pyogenes* Cas9, however to date there are 6 different CRISPR/Cas types, and at least 29 subtypes (206). Wild-type CRISPR/Cas9 is an endonuclease, and can be used to cut DNA and facilitate gene editing through either non-homologous end joining (NHEJ) or homology-directed repair (HDR). Modification of the Cas9 protein to be catalytically dead results in deactivated Cas9 (dCas9), which can then be fused to an array of transcriptional regulators, or epimodifers, to directly modulate the expression and/or context of virtually any endogenous gene. For example, fusion of the CRISPR/Cas protein with DNA methyltransferase enzymes DNMT3A and DNMT3B can be used to catalyze *de novo* DNA methylation, while fusion with ten-eleven translocation (TET) proteins can be used to specifically achieve DNA demethylation. Additional fusions can be made for transactivation (fusion with VP64, VPR), transrepression (KRAB), and locus-specific histone modifications, such as acetylation (p300), deacetylation (HDAC3), methylation (PRDM9) and demethylation (LSD1), to name a few examples (207). Adding to this, while first generation systems involve the fusion of a single effector domain directly to the dCas9 protein, the development of second-generation systems has enabled amplified modification through recruitment of multiple effector copies, in addition to spatial and temporal control of the modifications (207–209). First and second-generation systems are illustrated in Figure 5.

One particularly advantageous feature of CRISPR/Cas technology for this type of application is the ability to multiplex. ‘Multiplexing’ is the term given when numerous guide RNAs (gRNAs) or Cas enzymes are expressed simultaneously. Using multiple gRNAs to target one gene can often result in a synergistic effect, in addition to decreasing the level of off-target effects. While the use of multiple gRNAs to target different genes at once may enable more robust regulation of the complex gene expression patterns, and enable more efficient reprogramming. Currently, multiplexing has largely been used to identify and understand cell transformation processes (210,211), and has garnered only limited attention as a therapeutic strategy. Saunderson *et al.* demonstrated *de novo* DNAme by multiplexing to target a series of genes commonly methylated in breast cancer, using CRISPR/Cas9 fused to both DNMT3A and DNMT3L. This approach enabled long-term hypermethylation and gene silencing of *CDKN2A*, *RASSF1*, *HIC1* and *PTEN* in primary human myoepithelial cells. They were able to initiate aberrant cellular processes, increasing our understanding of DNAme-driven early changes in breast cancers (210). Finally, multiplexing by using several types of Cas enzymes can lead to robust epigenetic changes and gene regulation. For example, O’Geen *et al.* investigated requirements to achieve persistent repression via CRISPR/dCas9 transcriptional repression and epigenetic modifications. They demonstrated that while long-term repression (14 days) could be achieved using a combination of CRISPR/dCas9-KRAB and DNA methylation, locus-specific histone methylation through CRISPR/dCas9-EZH2 is required for robust, long-term silencing (212). Considering the complexity of aberrant gene expression networks, there has nevertheless been promising progress that may lead to future therapeutics for the treatment of breast cancers.

#### Application of CRISPR/Cas therapeutics for targeted gene regulation in breast cancers

Interestingly, there are far fewer studies looking at the delivery of catalytically dead CRISPR/dCas technology as a potential therapeutic in comparison to the active nuclease. Perhaps this is due to the complexity of the relationships between epigenetics and gene expression, the intricate orchestra of TF expression relationships themselves, different dosage windows, or even due to gene editing being ‘easier’ to assess. Despite this, there have been several studies, both *in vitro* and *in vivo*, which do demonstrate the therapeutic potential of this approach. In 2016, Choudhury and coworkers achieved targeted demethylation of the *BRCA1* promoter with CRISPR/dCas9 fused to the catalytic domain of TET1. Their work demonstrated transcriptional upregulation of the gene after demethylation, in addition to a reduction in observed cell growth. The study was conducted using the MCF7 breast adenocarcinoma cell line *in vitro*, using Lipofectamine LTX as the delivery agent. This proof-of-concept study demonstrates that upregulation of BRCA1 expression exerts a significant inhibitory effect on cell viability, with or without additional chemotherapeutic agents (25). Huang *et al.* used a light-inducible genetic circuit using the dCas9 system fused to VPR a transactivation domain to activate the expression of either p53 or E-cadherin. They developed an AND logic gate, where the circuit requites presence of both the human telomerase reverse transcriptase (hTERT) and human uroplakin II (hUPII) promoters to be activated. In this example, activation of p53 and E-cadherin had a significant effect on reduction of cell proliferation, invasion and apoptosis. While this particular study was conducted in a bladder cancer model, it serves as a good example of what can be achieved using dCas9 (213).

Rather than directly targeting TFs, which are notoriously difficult to drug, other studies have instead targeted upstream tumor suppressor genes. For example, *PTEN* (phosphatase and tensin homolog deleted from chromosome 10) is a critical tumor suppressor gene: *PTEN* expression negatively correlates with tumor size, pathological grade, and the expression of both ER and PR in breast cancer (47,214). PTEN expression is lost in up to 30% of sporadic breast tumors via DNA methylation of the PTEN promoter, and transcriptional repression of PTEN can lead to resistance to clinical treatments. Moses *et al.* used dCas9 fused to the transactivator VP64-p65pRta (VPR) to reactivate PTEN expression in triple negative breast cancer cell lines. This was achieved *in vitro* by first establishing cell lines stably expressing the dCas9-VPR system via lentiviral transduction, and then mixtures of gRNAs were transiently introduced using the commercial reagent Lipofectamine 2000. PTEN activation significantly repressed downstream oncogenic pathways, and suggested that combining CRISPR activation of PTEN with conventional small molecule inhibitors, such as PI3K/mTOR or B-Raf inhibitors, could increase the efficacy of tumor inhibition and limit potential of developing resistance (215). Liu *et al.* took a different approach and designed a polymeric nanoparticle for multistage delivery of CRISPR/dCas9 fused with a VP64 transactivator. The core construct of the nanoparticle includes phenylboronic acid (PBA) modified with low molecular weight polyethyleneimine (PEI), which interacts and condenses with the CRISPR/dCas9 DNA. The shell was then formed by 2,3-dimethylmaleic anhydride-modified poly(ethylene glycol)-*b*-poly(lysine), which is designed to dissociate within the acidic tumor environment. miR-524 is a microRNA that has been found to be suppressed in several types of cancer, including breast cancers. Restoring endogenous expression of miR-524 can suppress the proliferation and metastatic potential of cancer cells. Liu *et al.* demonstrated suppressed tumor growth both *in vitro* and *in vivo*, in a TNBC model (216). Finally, work in our group has demonstrated tumor suppression and regression through the reactivation of silenced tumor suppressor genes *MASPIN* and *CCN6* in an MCF7 xenograft mouse model of breast cancer. Expression of *MASPIN* was reactivated using CRISPR/dCas9-VPR together with a synergistic activation mediator (SAM) complex multiplexed with 4 gRNAs, while *CCN6* expression was reactivated using CRISPR/dCas9-VPR targeted via 5 gRNAs. This study utilized a cationic polymer which had been designed for targeted delivery by way of a cyclic RGD peptide (217).

Disruption of secondary structures using Cas9 is also possible, albeit the field is in its infancy and currently aimed at understanding the regulatory role of structures, rather than application as a therapeutic. So far, the active Cas9 nuclease has been utilized to create mutations within G4-forming sequences. Huang *et al.* used this approach to disrupt the G4 structure involved in the regulation of the chloride intracellular channel 4 (*CLIC4*) gene, which has been found to have tumor-promoting properties. It was found that the transcription activity of CLIC4 decreased upon G4 disruption (218). Li *et al.* utilized Cas9 targeting G4 forming sequences to elicit the relationship between TF YY1-mediated DNA looping in a G4-dependent fashion. Disrupting the formation capability of specific G4 structures was found to result in the significant reduction of YY1 binding and DNA looping (182). Together, these examples begin to demonstrate the potential of combining the precise targeting capability of systems such as CRISPR and the unique genomic distribution and structure of G4/i-motif regulatory elements.

It is clear, however, that examples are somewhat limited, which may arise from several contributing reasons. Adding to the complexity of regulating gene expression patterns is that modulating gene expression is not always the only objective—the gene must be expressed in the correct context. For example, *OCT4*, a master transcription factor of pluripotency, can act as both a tumor suppressor gene or an oncogene, depending on the cellular context (219). MASPIN is another example, where localization of the expressed protein dictates its role as a tumor suppressor (220).

#### Hurdles in the administration of CRISPR/Cas

Perhaps the biggest obstacle that CRISPR/Cas therapeutics face is the method of delivery (221). One of the main reasons for this issue is the size of the CRISPR/Cas construct, in addition to the requirement of simultaneous delivery of multiple components (222): the CRISPR/Cas, one or more short guide RNA (gRNA), and the effector domain (for epigenetic editing or transcriptional modulation) or template DNA, in the case of homology-directed editing. Transfecting large and multiple constructs results in a statistical distribution, where many cells do not receive all components (223). CRISPR/Cas therapeutics can be delivered in the form of RNA, DNA or protein, and can be delivered so as to achieve sustained expression, or as a transient ‘hit and run’ approach (224). These factors all affect the end dosage, the therapeutic window, and potential for off-target effects. Further, the target location within the body also impacts the choice of delivery method. For breast cancer, and other solid tumors, systemic delivery would be more clinically applicable than intracellular delivery methods such as microinjection or electroporation (222).

Gene delivery methods can be broadly divided into viral and non-viral delivery. Viral delivery involves the usage of engineered viruses, such as lentiviruses or adeno-associated viruses (AAVs), to deliver the therapeutic cargo. Viral delivery methods still face problems due to toxicity and immunological concerns (both initial and adaptive), limited intrinsic packaging capacity, and cost. Lentiviruses have a high carrying capacity of ∼8 kb; however, they cause integration into the host genome which not only risks insertional mutagenesis but also causes long-term expression, which inherently increases the chances of off-target effects and toxicity when delivering CRISPR/Cas (225–227). AAVs are more favorable for gene delivery, as the chance of integration is low. However, AAVs struggle to encapsulate the CRISPR/Cas sequence as they are limited to a carrying capacity of ∼4.7 kb. Smaller variants of the CRISPR/Cas have been developed (228), such as *Staphylococcus aureus* Cas9 (*Sa*Cas9). Nevertheless, for epigenetic or transcriptional applications, further space is required for the expression of the additional effectors, and so even the smaller orthologs can exceed the AAV carrying capacity (229). AAV-based delivery methods may be rendered ineffective by immune activation, as AAVs are common and 40–80% of adults will already have AAV antibodies. Therefore, patients often have to take immune suppressants in addition to the AAV therapeutic, and repeat dosing becomes a challenge, although there has been promising development of immune-orthogonal orthologues of AAV capsids to overcome this issue (230). Finally, AAV-based therapeutics hold the record for being the most expensive drugs available, and therefore are currently not applicable for treating something as widespread as breast cancer. Glybera, for example, an AAV-based therapeutic aimed at treating a rare inherited lipoprotein lipase deficiency, costs US$1.2 million per patient (231).

On the other hand, non-viral delivery encompasses all other methods of delivery, including physical methods such as electroporation, microinjection, hydrodynamic delivery and sonoporation, and synthetic delivery, such as with lipids, polymers and inorganic nanoparticles. While typically the delivery efficiency does decrease with large therapeutic cargoes, non-viral methods do not face a restricting size limitation, unlike viral delivery. All non-viral delivery methods are transient in nature, which lowers the chance of off-target effects (232). However, it also means that the therapy delivered must either be fast-acting, or create stable and inheritable changes within the cell. Some non-viral delivery methods such as highly cationic or PEGylated agents, have demonstrated potential toxicity or immunological responses (233,234), but the high variety in potential chemical design inherently enables enough flexibility to overcome these issues (227). The most limiting factor faced by non-viral delivery methods is simply that the delivery efficiency is not as high as their viral counterparts, especially when delivering to particular cell types, such as post-mitotic cells. Another challenge is achieving sufficient tumor targeting and penetration depth within highly dense and heterogeneous tumors, which has been a challenge even for promising delivery vehicles such as nanoparticles (235).

### Summary

Targeting cancer progression at the genetic level has not been previously achievable but may be realized through the application of either small molecules that target DNA secondary structures or more flexible and advanced CRISPR/Cas9 technology. Small molecules capable of stabilizing G4 or i-motif structures show good promise as a therapeutic approach. However, the level of specificity that is attainable for these highly varied and ubiquitous structures, and only within target cells, is still uncertain. CRISPR/Cas9 technology may provide an alternative approach, enabling specific gene editing or regulation through transactivators/repressors and epigenetic enzymes. Off-target delivery could potentially be minimized or avoided through sophisticated inducible Cas9 systems, and specific cell-type targeting. Since EMT-TFs act in concert, targeting just one gene at a time may not lead to robust gene expression and substantial phenotypic changes. Multiplexing to target multiple genes at once may solve this problem. Additionally, exploiting the heritability of epigenetic marks such as DNAme may assist in achieving a sustained effect even after transient treatment. One of the biggest limitations to the implementation of CRISPR/Cas9 technology is the difficulty of efficient and safe delivery.

## CONCLUSION AND FUTURE OUTLOOK

Breast cancer is a highly heterogeneous and complex disease, with individual patients presenting unique profiles brought about by mutations, gene silencing or activation, and past therapy. Linking each of these profiles is anomalous gene expression, which can, in turn, alter TF interactions and in a feedback loop, alter the expression of other genes. This dysregulation of gene expression contributes to the hallmarks of cancer and processes such as EMT through alterations in TF binding, chromatin organization and epigenetic marks. Ultimately, disruption of normal patterns of gene expression contributes to recurrence and metastasis, which represent the principal cause of death in breast cancer patients today. Correcting and gaining control over aberrant gene expression therefore represents a highly attractive yet extremely complex therapeutic opportunity in breast cancer. However, as it stands for now, we still lack a coherent overview of the molecular and biochemical mechanisms inducing cells to enter various states along the epithelial–mesenchymal phenotypic spectrum and an improved understanding of the dynamic and plastic nature of the EMT program is required to fully utilize the therapeutic potential within this space.

As with all therapies, specificity will be key. Tight regulation of gene expression is crucial for normal cell processes and health, and so any therapeutic strategies to correct aberrant TF expression, disrupted TF binding, or abnormal secondary structure formation must not impact healthy cells and tissue. In this respect, small molecules designed to stabilize G4 and i-motif structures face a formidable challenge in terms of specificity and selectivity. With >700 000 potential G4 sites in the human genome and some 10 000 detected in chromatin (74), it will no doubt be challenging to design drugs that are highly specific for a given G4 or i-motif target. Furthermore, the complexity of TF signaling pathways complicates potential therapies designed to modulate DNA–protein interactions, as they may be highly interdependent. Although individual studies look at knockdown of individual TFs, further research to untangle the roles of individual TFs will be needed, which may require further development of multiplex or high-throughput methods such as CRISPR-based screening (236–239).

Targeting G4 stability through DNAme may present a potential alternative to small molecules. For example, regulating the formation of G4s or i-motifs may be possible through the utilization of CRISPR/Cas9 to methylate or demethylate particular loci. New therapeutic approaches will appear as our understanding of the relationship between DNA secondary structures, epigenetic modifications and gene expression increases, which may lead to robust gene regulation and EMP ‘resetting’ of carcinoma cells through CRISPR/Cas technology. However, before CRISPR/Cas technology can be utilized as a cancer therapeutic, the development of non-viral gene delivery is necessary and multidisciplinary collaborations between labs in these fields is crucial. Transient delivery of CRISPR remains a bottleneck associated with low therapeutic efficiency and hindered tumor penetration. Further research into the development of delivery agents, including in 3D culture to better model difficulties in delivery, will assist. In the future, personalized medicine will enable thorough profiling of each patients’ mutations, epigenome and transcriptome, rather than diagnosis based on a few select markers. Having a complete tumour profile will allow for more accurate diagnoses, including a more precise determination of breast cancer subtype, and the identification of aberrant expression patterns that should be corrected. Such personalized approaches may also help to overcome bias in clinical, pathological and genetic cancer data, which are skewed toward European populations (240). Targeting particular genes or pathways through G4 or i-motif secondary structure, or with CRISPR-based technology, will offer highly specific approaches to therapy, including re-activation of silenced genes or successful interventions against undruggable targets.

Overall, there are many opportunities on the horizon that will enable gene-specific, or gene-pathway-specific, treatments for breast cancers. Targeting the aberrant expression of genes involved in cancer progression, metastasis, and development of resistance through EMT has the potential to yield novel therapies and improved management of high-grade malignancies.
